# Biomimetic antimicrobial cloak by graphene-oxide agar hydrogel

**DOI:** 10.1038/s41598-016-0010-7

**Published:** 2016-12-05

**Authors:** Massimiliano Papi, Valentina Palmieri, Francesca Bugli, Marco De Spirito, Maurizio Sanguinetti, Carlotta Ciancico, Maria Chiara Braidotti, Silvia Gentilini, Luca Angelani, Claudio Conti

**Affiliations:** 1 Physics Institute, Catholic University of the Sacred Heart (UCSC), Largo Francesco Vito 1, 00168 Rome, Italy; 2 Institute for Complex Systems, National Research Council (ISC-CNR), Via dei Taurini 19, 00185 Rome, Italy; 3 Microbiology Institute, Catholic University of the Sacred Heart (UCSC), Largo Francesco Vito 1, 00168 Rome, Italy; 4 Department of Physics, University Sapienza, Piazzale Aldo Moro 5, 00185 Rome, Italy; 5 Department of Physical and Chemical Sciences, University of L’Aquila, Via Vetoio 10, I-67010 L’Aquila, Italy

## Abstract

Antibacterial surfaces have an enormous economic and social impact on the worldwide technological fight against diseases. However, bacteria develop resistance and coatings are often not uniform and not stable in time. The challenge is finding an antibacterial coating that is biocompatible, cost-effective, not toxic, and spreadable over large and irregular surfaces. Here we demonstrate an antibacterial cloak by laser printing of graphene oxide hydrogels mimicking the Cancer Pagurus carapace. We observe up to 90% reduction of bacteria cells. This cloak exploits natural surface patterns evolved to resist to microorganisms infection, and the antimicrobial efficacy of graphene oxide. Cell integrity analysis by scanning electron microscopy and nucleic acids release show bacteriostatic and bactericidal effect. Nucleic acids release demonstrates microorganism cutting, and microscopy reveals cells wrapped by the laser treated gel. A theoretical active matter model confirms our findings. The employment of biomimetic graphene oxide gels opens unique possibilities to decrease infections in biomedical applications and chirurgical equipment; our antibiotic-free approach, based on the geometric reduction of microbial adhesion and the mechanical action of Graphene Oxide sheets, is potentially not affected by bacterial resistance.

## Introduction

Population aging and advances in materials technology increase the usage of biomaterials and medical devices^1–4^. Research in this field focuses on the microorganism colonization on devices and the resulting biofilm formation^1, 5–7^, which adversely affect the implant and cause systemic effects on the patient. Broad-spectrum antibiotics are an effective prophylaxis strategy, but the therapy is often toxic and leads to the appearance of multi-drug resistant microorganisms^2^.

For impeding bacterial colonization, there is the need of finding covering strategies not harmful to humans, cost-effective and with long lasting effects^8^. Substances exploited for coating include antibiotics^9^, silver^10^, titanium^11^, hydroxyapatite^12^, and the fluoride ion^13^. For a prolonged effect, the treatment must be repeated^14, 15^ with severe cumulative toxic effects on patients^16^.

Graphene Oxide (GO) has antimicrobial effects causing membrane disruption, bacteria wrapping, and induction of oxidative stress^17, 18^. GO is environment-friendly and presents mild cytotoxicity to mammalian and plant cells. Compared to other carbon nanomaterials, the easy processing, large-scale production, and inexpensive cost guarantee the GO can be a good new antibacterial agent. Together with the application potential, the advantages of inhibiting or killing bacteria by graphene oxide include unique features compared to other antibacterial materials or agents, like silver. One of the most often observed effects is the bacteria cutting by GO sharp edges. This is known as the nano-knife or nano-blade effect. GO blades cut the cell membrane and cause the leakage of intracellular constituents to the environment and leads to microorganisms death. GO also induces oxidative stress interfering with cellular metabolism and cell necrosis/apoptosis. Oxidative stress mainly comes from two pathways: ROS-dependent or ROS-independent oxidative stress. The former is due to the accumulation of intracellular ROS by the adsorption of O_2_ on defect sites and edges of GO, followed by reduction by various enzymes like glutathione. The ROS-independent stress is caused by to the oxidization of vital cellular structures by charge transfer from cellular membrane to graphene oxide that acts as an electron pump. Finally, a third mechanism is the isolation of bacteria from external environment: in the wrapping/trapping, the sheets form a blanket over bacteria and insulate them from nutrients^17^.

Several groups incorporated GO into hydrogels for antimicrobial use with easiness of preparation, high efficiency, and cost-effectiveness^19, 20^. In the GO hydrogels production, a GO solution is mixed with a “gelator”, like cellulose^21^, chitosan^22^, elastin^23^, which accounts for the material three-dimensional structure. However, the bulk of the material entraps GO minimizing the contact surface between GO sheets and bacterial cells.

A further low-toxicity antimicrobial strategy is based on bio-inspired surfaces that mimic real animal features, engineered by evolution, with very effective antibacterial action due their specific roughness and shape^24^. Examples of natural antibacterial surfaces include the shark skin, the dragonfly wings or the Cancer Pagurus carapace^1, 3^. However, a precise surface engineering to reproduce these natural structures may be not feasible for arbitrary shape medical tools and very limited for hydrogels.

Our idea is to combine the GO and a natural antibacterial pattern in a way that amplifies their specific advantages while overcoming the described limitations.

We entrap GO in a gelified material (agar) and shape the circular surface topography of the Cancer Pagurus carapace combined with a hierarchically wrinkled surface. The abundant –OH groups in agar have strong interactions with the functional groups on GO avoiding the shrinkage and aggregation of the GO nanosheets. To shape the hydrogel, we use a novel effect discovered in nonlinear optics: the laser induced supercavitation^25^. During the laser supercavitation, the gel is locally liquefied by the laser pulse and the temperature gradient produce an explosive expansion that removes the surface. The mechanism is a combination of evaporation and thermally induced transport. With high-power laser pulses we locally ablate the soft material, exposing GO nanosheets cast in the polymer. By this approach, we laser print the bio-inspired morphology.

**Figure 1:**
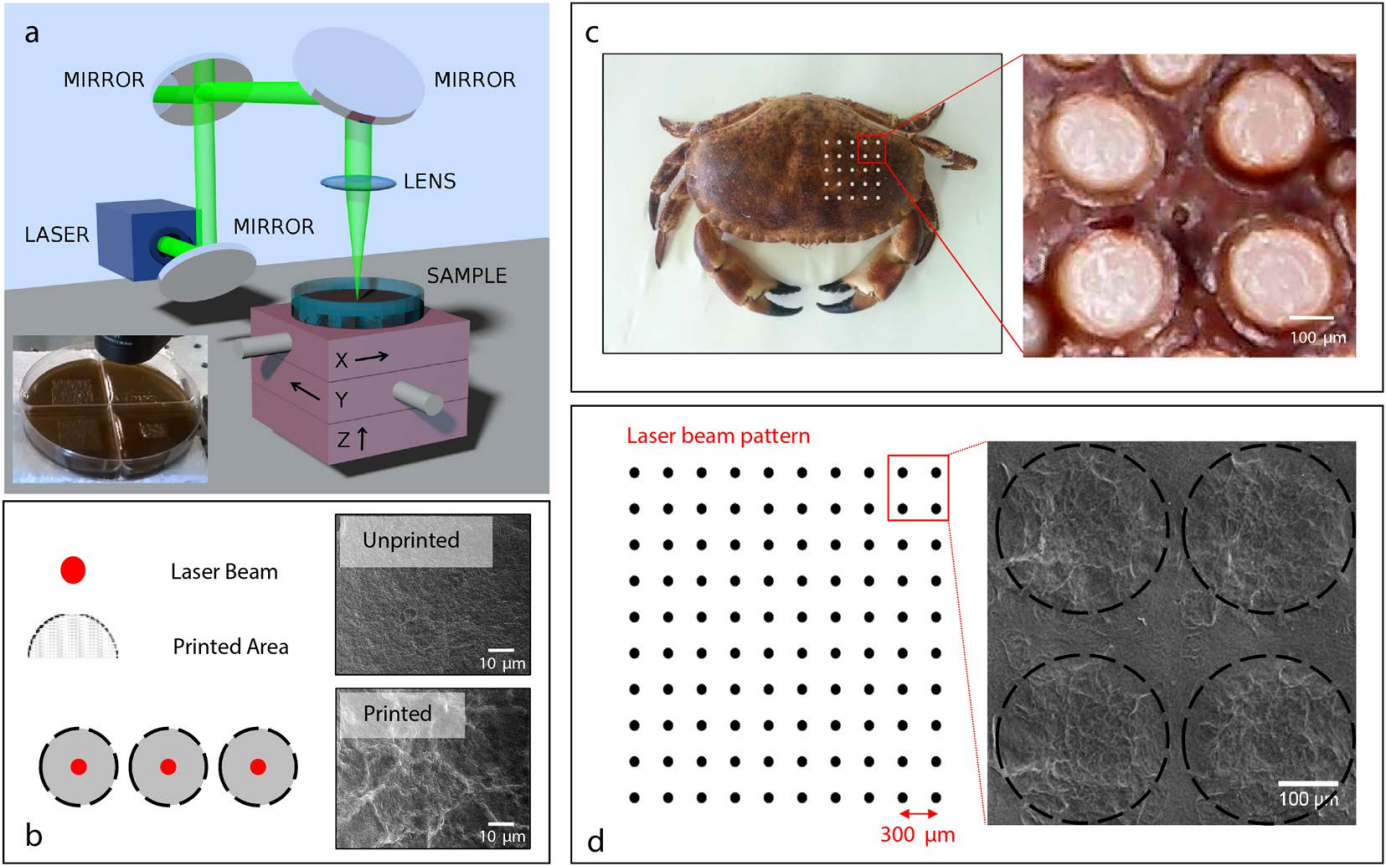
Pattern realization. (**a**) Experimental setup for the laser printing of the surface of the Agar substrates. Inset: Patterned GO-agar substrate by focusing a 5 mJ laser pulse. (**b**) On the left, sketch of the laser pulse action as occurs on the GO-based substrate: the red bullets represent the point interested by the laser pulse, the gray area is subject to the cavitation induced by the thermal expansion of the GO-Agar. The panels on the right show SEM images of the unprinted (top) and printed (bottom) area of the substrate (Figure S1 shows further details). (**c**) Cancer Pagurus (left) and detail of the carapace pattern (right). (**d**) Sketch of the geometry of the optically realized patterns (left) and SEM images (right).

In Fig. 1a, we report the experimental setup used to realize the laser printing. The displacement in the x-y plane allows realizing the desired pattern, while the displacement along the z-axis controls the focus position and, hence, the size of the removed material area. Figure 1a inset shows a typical patterned GO substrate.

Figure 1b shows the excavated regions. We design the experimental parameters, as laser spot, pulse energy, and irradiation pattern to mimic the wrinkled surface of the Cancer Pagurus that sustains unexpected and very effective antibacterial actions^3^ (see Fig. 1c). The size of the larger excavated region the laser energy is correspondent to the size of the large feature of the carapace (250 μm). Also, the supercavitation produces a wrinkled surface with thinner features which are surprisingly similar to the natural surface of the Cancer Pagurus.

By using this approach, we realize substrates, as sketched in Fig. 1d, with alternating laser treated regions and blank regions. On these substrates, we grow human pathogens E. coli, S. aureus, and C. albicans.

**Figure 2:**
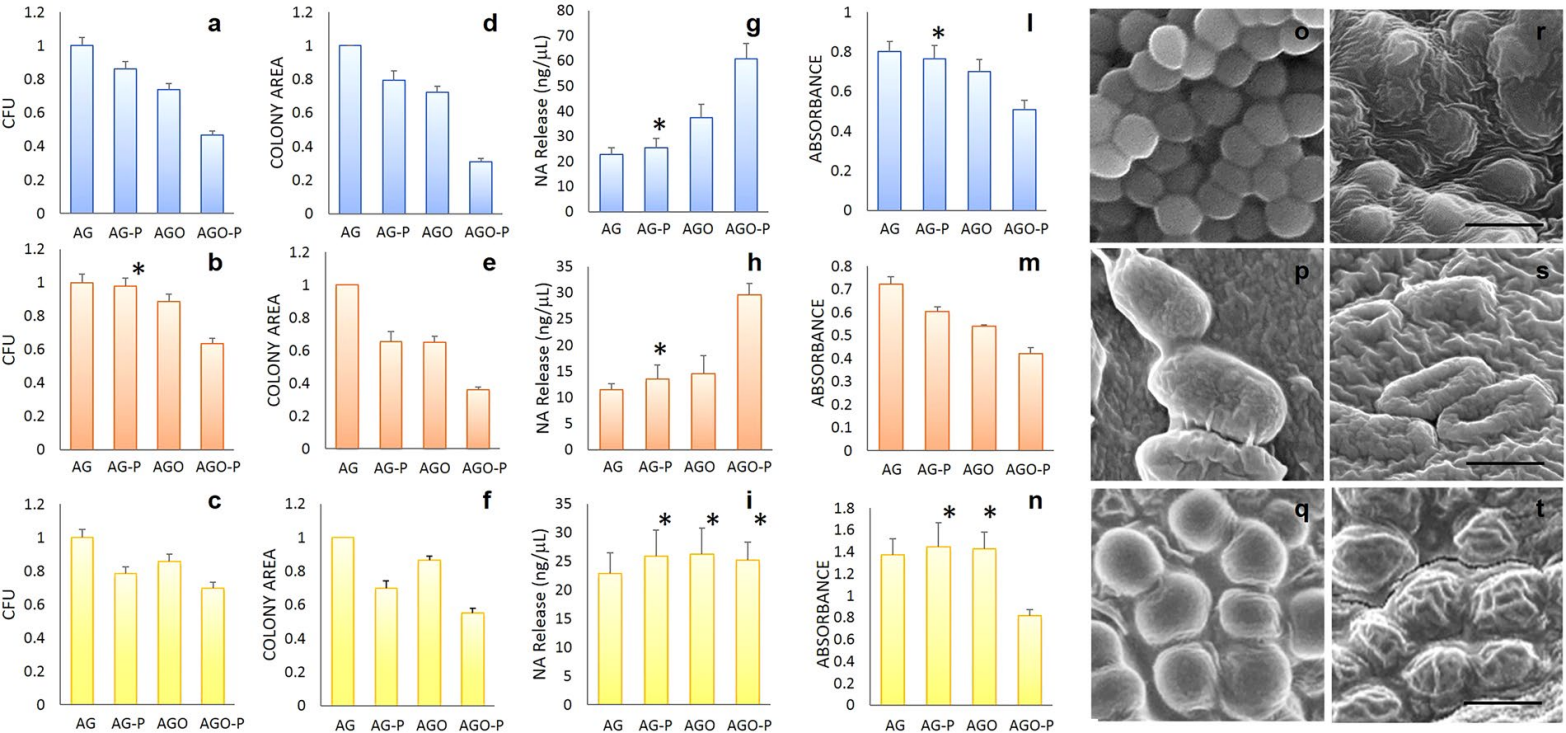
CFUs number, colony size, cell damage, metabolic activity and structural integrity of microorganisms grown on different substrates. Number of CFUs on different hydrogels: S. Aureus (**a**), E. Coli (**b**) and C. Albicans (**c**). Normalized colony diameter on different hydrogels: S. Aureus (**d**), E. Coli (**e**) and C. Albicans (**f**). Nucleic acid released after exposure to different hydrogels of S. Aureus (**g**), E. Coli (**h**) and C.Albicans cells (**i**). Metabolic activity quantification using XTT test for S.Aureus (**l**), E. Coli (**m**) and C.Albicans cells (**n**). Representative Scanning Electron Microscopy images of for S.Aureus (**o**), E. Coli (**p**) and C.Albicans (**q**) on AGO hydrogels or AGO-P hydrogels (**r–t**). Scale bar is 1 μm in (**r**) and (**s**) and 10 μm in (**t**). Asterisks indicate statistically not significant differences compared to the untreated agar hydrogel AG.

Firstly, we evaluate the number of colony-forming units (CFUs) and colonies size (Fig. 2a–c) on Agar (AG), laser printed Agar (AG-P), Agar mixed with GO **(**AGO), and laser printed Agar mixed with GO (AGO-P).

The CFUs on AG-P displays a 10% decrease for all species considered. The CFUs on AGO are reduced by a similar amount. On the other hand, the AGO-P causes a marked reduction of CFUs: 53% for S. aureus, 40% for E. coli and 30% for C. albicans.

Surface patterning alters the microorganism growth rate and consequently the colony size (Fig. 2d–f). Colonies size slightly decreases on AG-P and AGO. The most evident effect is visible on AGO-P where S. aureus displays a colony area reduced by about 70%, E. coli is reduced by 65%, and C. albicans colonies are 45% smaller.

According to these results, we can claim that the gram-positive S. aureus is more sensitive to GO due to the lack of outer membrane, external to the peptidoglycan layer, which is protective for Gram-negative E. coli. Finally, the GO effect is less visible in C. albicans fungus that possesses a thick and complex cell wall structure compared to bacteria^26, 27^.

We address the cell damage by measuring the amount of Nucleic Acids (NA) released after cell disruption (Fig. 2g–i). Concentrations of NA released by bacteria exposed to AGO-P are meaningfully higher than those released after exposure to all the other hydrogels. This evidence shows that GO nanosheets, exposed after hydrogel patterning cut as nano-knives the bacterial membrane (see Fig. S1). Differently, an increase of NA release is not visible for C. albicans, which as discussed above, possess a robust cell wall (Fig. 2i).

XTT results (Fig. 2l–n) clearly indicate that cell proliferation is slowed down for all species on AGO-P. Only a small impairment of bacteria metabolism is visible on AG-P and AGO. On the contrary, C. albicans metabolic activity is not affected on these surfaces, but only on AGO-P surfaces.

For all species on the AGO-P, SEM images show cellular membrane morphological changes and collapse of cell structure embedded into the wrinkled surface (Fig. 2o–t).

**Figure 3:**
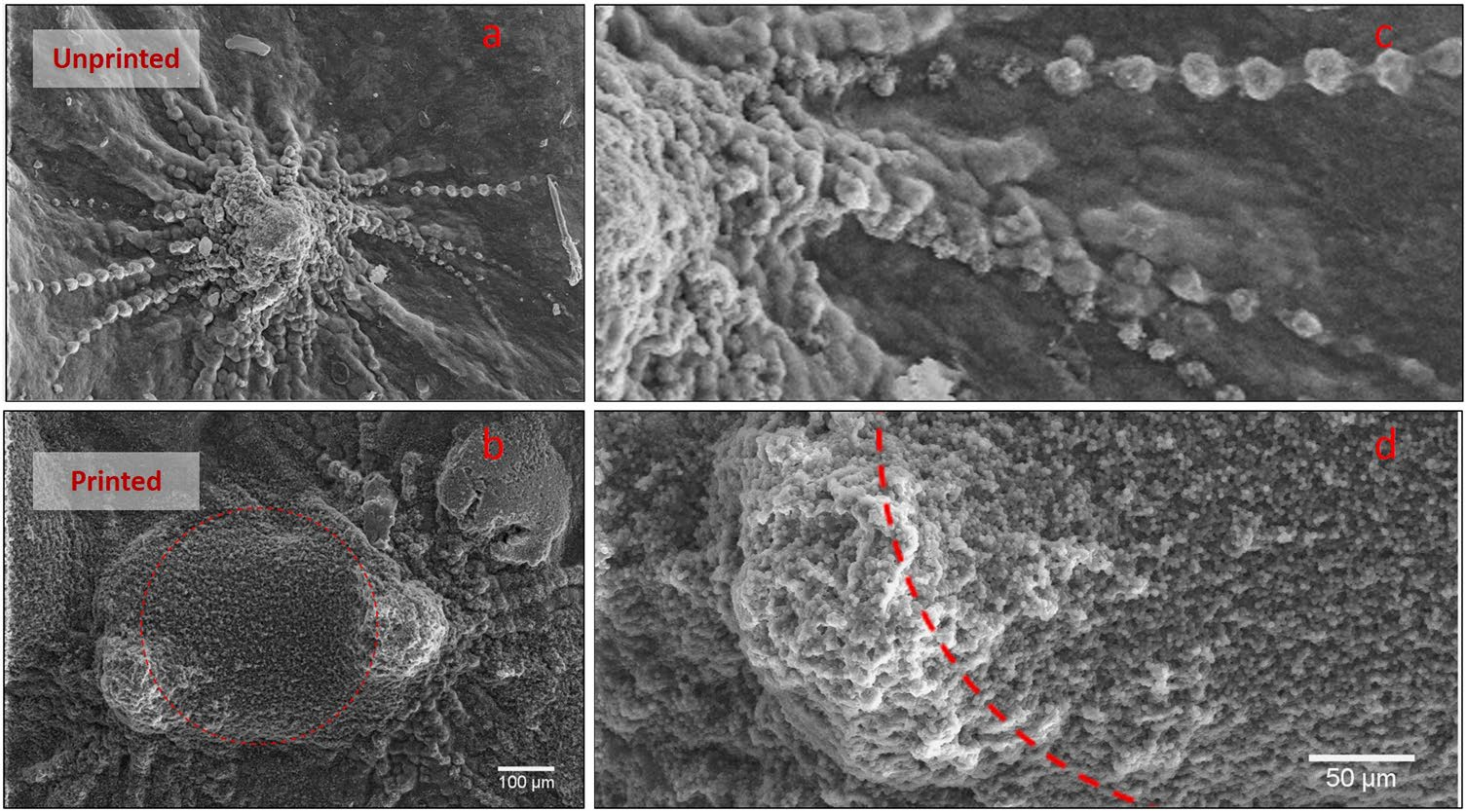
C. Albicans morphology. Image of hyphae initial formation on AG (**a,c**) and AGO-P (**b,d**). Dashed red lines represent the bleached areas of the hydrogel. When C. Albicans colonies meet the pattern on AGO-P, cells lose their ordered structure completely and fill the circular holes.

A combination of bactericidal and bacteriostatic effects is then the key advantage in using GO patterned hydrogel. Firstly sharp GO sheets, exposed by laser ablation, cut bacterial membranes and leads to nucleic acids leakage. Secondly, GO embedded in wrinkled structures reduces cell metabolism. The GO edges do not cut the fungus protected by a thicker cell wall. However, the fungus is still sensitive to the bio-inspired pattern that slows the colony growth and cell metabolism. As shown in Fig. 3, the wrinkled AGO-P surface, able to trap the fungus cells, overshadows the planned colony program and does not allow the formation of the typical hypha elongated structure.

To model the microorganisms growth in a complex pattern, we develop a two-dimensional off-lattice simple model for the cell growth and duplication. A spherocylinder of fixed diameter *a* and variable length ℓ (the initial length is ℓ_0_) represents each cell. During the growing process the cell length ℓ increases at constant rate. At ℓ = 2ℓ_0_ the cell is replaced by two equal cells of length ℓ_0_. Repulsive inter-cells forces mimic excluded volume effects. We switch-off the cell growing in the irradiated regions to modelling the bactericidal properties. Moreover, we include partial cells overlap in the laser irradiated killing regions to account for additional available space (further details in Methods).

**Figure 4:**
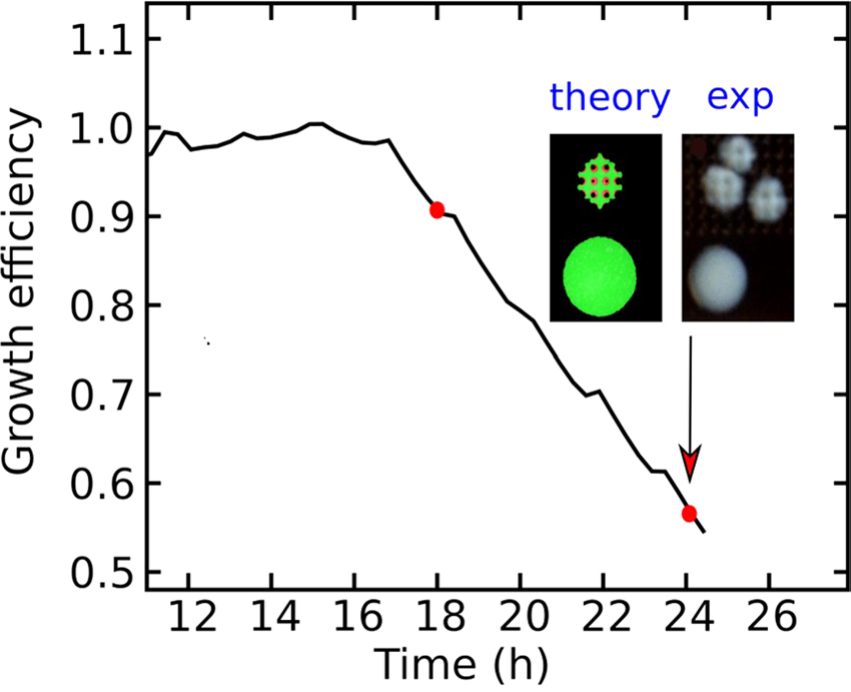
Comparison with the theoretical model. Numerical calculation (thick black line) of the growth efficiency and comparison with experimental results (red dots). The insets show growing cells in an unstructured (bottom picture) and structured (top picture) environment. The experimental results in the insets refer to E. Coli after 24 h.

Figure 4 shows the agreement between the experimental results and the theoretical analysis. The “growth efficiency”, the ratio between the colony size in the pattern surface and the colony size in the unpatterned case, goes from 0 for a total ideal duplication inhibition to 1 in the absence of any antibacterial action. Growth efficiency reduces with time because the colonies explore a growing antibacterial region. Insets in Fig. 4 show the shape of E.coli colonies after 24 h on AGO-P and AGO, compared with the snapshot of the simulation of a colony obtained starting from a single initial cell duplicating in a sculptured surface. The model agrees with the experimental results using the temporal step for replication as a fitting parameter.

Nowadays, new infectious organisms, built by selected genetic mutations, display an impressive drug resistance. Microorganisms learn very fast to resist to antibiotics by changing metabolic pathways or modify specific antibiotic molecular targets.

The pressing global query is antimicrobial coatings that may potentially revolutionize the health care industry for nosocomial infections, especially for immunocompromised patients or low-income countries. Among the novel strategies, the idea of mimicking non-toxic natural fouling defense mechanisms represents a very promising scenario.

We realize an effective hydrogel cloak with a broad spectrum of antimicrobial activity by a novel laser printing technology. We obtain a bacterial reduction up to 53% in CFU and 70% in size; this corresponds to cut the cells by nearly 90%.

We combine GO natural antimicrobial effect with a bio-inspired pattern having multiscale wrinkles. These two antimicrobial actions drastically enhance their efficacy when joined. Considering that the GO action against microorganisms is mainly due to a mechanical disruption of cell walls and not related to a specific site of interaction or drug accumulation inside the bacteria, this mechanism is potentially unaffected by the development of resistance.

## Methods summary

We realized a Graphene Oxide-Based hydrogel, by mixing GO solution (1 mg/ml) with Luria Bertani agar powder (2%) in ultrapure water. GO and agar solution gelation occurs in a temperature dependent manner, without involving any organic solvent. For laser patterning, we employ a frequency-doubled Q-switched Nd: YAG pulsed laser (λ = 532 nm), with 10 Hz repetition rate and 6 ns pulse duration. We analyze Growth and metabolism of Gram-positive bacterium S. aureus, Gram-negative E. coli, and C. albicans fungi on hydrogels by Colonies Forming Units Assay, Nucleic Acids Release, and XTT assay. We adopt optical and scanning electron microscopy for the morphology of colonies. The statistical significance of differences in mean values of all the parameters measured was assessed with the two-tailed Welch’s t-test. All statistical analyses were performed with the statistical software package STATA version 12.0 (Stata Corporation, College Station, TX, USA). p < 0.05 were considered statistically significant. We use an individual-based off-lattice model for the theoretical colony growth curves compared to experimental results.

## Methods

### Preparation of GO-Based Hydrogels

Autoclaved LB-Agar solutions have been mixed with GO sterile solutions to a final GO concentration of 1 mg/ml until a uniform suspension has been obtained. The mixture was poured in plates and let dry. LB-Agar plates without GO were used as control.

### Laser Printing

We use a frequency-doubled Q-switched Nd: YAG pulsed laser emitting at 532 nm wavelength, with 10 Hz repetition rate and 6 ns pulse duration, the explored energy power range between 2 to 10 mJ. We focus the laser beam on the Agar substrate by means the lens. The sample is placed on a three-dimensional translational stage to move it up to tens of millimeters with a precision of 100 nm.

### Microorganisms Growth on hydrogel surfaces

E.Coli (ATCC strain 25922), S. Aureus (ATCC strain 29213) and C. Albicans (ATCC strain 90028) have been used for the experiments. Cells have been maintained on MacConkey, Trypticase Soy with 5% Sheep Blood and BCG agar plates respectively. Microorganisms were resuspended in fresh PBS solution prior each experiment. The CFUs have been quantified by plating of serial 10^6^-fold diluted solutions in order to calculate the optimal number of microorganism per gel area for further experiments. 15 µl of bacteria/fungi solutions containing 10^3 ^CFU/ml have been plated per each cm^2^ of surface.

Bacteria/fungi on hydrogels have been incubated for 18 and 24 hours at 37 °C, and CFUs were quantified. Colony morphologies have been analyzed by using ImageJ software.

### Scanning Electron Microscopy

Samples for Scanning Electron Microscopy have been prepared as described elsewhere^28^ with slight modifications. Briefly, hydrogels have been fixated in Glutaraldehyde solution (2.5%) for 2 hours and then dehydrated serially in ethanol. After drying, the samples were sputtered with gold and micrographs were acquired using a Supra 25 microscope (Zeiss, Germany).

### Antimicrobial effect of GO hydrogels (Nucleic acid release quantification and XTT Assay)

To quantify cell damage, the release of intracellular nucleic acids has been measured by using UV absorption at 260 nm. A pool of 10 colonies grown on different surfaces has been re-suspended in fresh ddH_2_O and centrifuged at 2000 rev/min for 10 min to separate cells from the supernatant containing free DNA/RNA^27^. Results have been normalized for Optical Density values of the uncentrifuged samples to account for different colony sizes.

Metabolic activity of microorganism has been quantified by XTT viability assay. A pool of colonies grown on different materials has been resuspended in fresh ddH_2_O until a Mcfarland Turbidity of 0.5 was obtained. Serial dilutions of cells have been seeded in a 96- well in triplicate and after addition of XTT solution, incubated for 4 hours at 37 °C. A plate reader (IMark, Bio-Rad Laboratories Inc, Hercules, CA, USA) has been used to measure the absorbance at 475 nm and 660 nm (non-specific signal). The specific absorbance A of the sample has been calculated as follows:

1 $${{\rm{A}}}_{475{\rm{nm}}}({\rm{Test}})\mbox{--}{{\rm{A}}}_{475{\rm{nm}}}({\rm{Blank}})\mbox{--}{{\rm{A}}}_{660{\rm{nm}}}({\rm{Test}}).$$

### Growing cell simulation model

We simulate a colony of growing cells in a two-dimensional space using an individual-based off-lattice model^29^. Each cell *i* is described by the center of mass position **r**_*i*_ and the orientation vector **e**_*i*_ = ℓ_*i*_ **ê**_*i*_, where ℓ_*i*_ is the cell length and the unit vector **ê**_*i*_ denotes the cell growing direction. We call ℓ_0_ the length at rest of the cell and *a* its thickness (the cell aspect ratio is *α* = *a*/ℓ_0_). The cell length ℓ_*i*_ is considered variable: during the cell growth, it increases from the rest value ℓ_0_ to 2ℓ_0_ at constant rate γ. When the *i*-th cell has doubled its length it splits into two equal cells *i*_1_ and *i*_2_ of length ℓ_0_, located at ${{\bf{r}}}_{{i}_{1},{i}_{2}}={{\bf{r}}}_{i}\pm {\hat{{\bf{e}}}}_{i}{\ell }_{0}/4$ and with orientation ${{\bf{e}}}_{{i}_{1},{i}_{2}}={\ell }_{0} {\mathcal R}$[**ê**_*i*_]. The operator ℛ performs a small random rotation of an angle *η*, which takes into account the effects of noise and prevents the formation of a long file of aligned cells during the replication process starting from a single cell.The equations of motion for the *i*-th cell are:

2 $$\,{{\boldsymbol{V}}}_{i}={{\boldsymbol{M}}}_{i}\cdot {{\boldsymbol{F}}}_{i}$$

3 $${{\boldsymbol{\Omega }}}_{i}=\,{{\bf{K}}}_{i}\cdot {{\boldsymbol{T}}}_{i}$$

where ***V***_*i*_ and **Ω**_*i*_ are the translational and rotational velocities, ***F***_*i*_ and ***T***_*i*_ the force and torque acting on the cell, ***M***_*i*_ = ***m***_||_ ***ê***_*i*_ ***ê***_*i*_ + ***m***_⊥_(1−***ê***_*i*_ ***ê***_*i*_) and ***K***_*i*_ = ***k***_||_ ***ê***_*i*_ ***ê***_*i*_ + ***k***_⊥_(1−***ê***_*i*_ ***ê***_*i*_) the mobility matrices, with ***m***_||,⊥_ and ***k***_||,⊥_ the parallel and transverse translational (***m***) and rotational (*k*) cell mobilities^29^. Mobility parameters depend on cell shape (length and thickness), and we determine them using analytic expressions for prolate spheroids^30^. We denote with *m*_0_ the translational mobility of the cell at rest, *m*_0_ = *m*_||_(ℓ_0_). We consider here steric pair repulsion forces acting along the minimum distance vector ${{\boldsymbol{r}}}_{ij}^{(min)}$ between the two orientation vectors ***e***_*i*_ and ***e***_*j*_ of each couple of cells *i* and *j*. The total force on the *i*-th cell is ${{\bf{F}}}_{i}=\sum _{{\rm{j}}\ne {\rm{i}}}{\bf{f}}({{\bf{r}}}_{ij}^{({\rm{\min }})})$. We chose for the pair force a Lennard-Jones truncated form, i.e. **f**(**r**) = f_0_[(*a*/r)^13^−(*a*/r)^7^]$\hat{{\bf{r}}}$ for r < *a* and 0 otherwise. We use the following values in the simulations: *α* = 1/2, *η* = 3·10^−3^ *π*, *γ* = 1 (internal units: ℓ_0_ for length, f_0_ for force, *m*_0_ for mobility). The equations of motion (1) and (2) are numerically integrated by the Runge-Kutta method (using a time step of 5·10^−6^).

To account for the bactericidal effects of the laser treated surface we use effective local parameters describing cells activity and motion. We consider that, inside a bactericidal region, the cells: do not replicate (we set *γ* = 0), can partially overlap (steric forces are reduced by a factor 10^−4^), get trapped due to cloak effects of the surface (mobility parameters are reduced by a factor 5·10^−2^).

## Electronic supplementary material

Supplementary Information
