# Erratum: Biomimetic antimicrobial cloak by graphene-oxide agar hydrogel

**DOI:** 10.1038/s41598-017-00063-5

**Published:** 2017-06-01

**Authors:** Massimiliano Papi, Valentina Palmieri, Francesca Bugli, Marco De Spirito, Maurizio Sanguinetti, Carlotta Ciancico, Maria Chiara Braidotti, Silvia Gentilini, Luca Angelani, Claudio Conti

**Affiliations:** 1 Physics Institute, Catholic University of the Sacred Heart (UCSC), Largo Francesco Vito 1, 00168 Rome, Italy; 2 Institute for Complex Systems, National Research Council (ISC-CNR), Via dei Taurini 19, 00185 Rome, Italy; 3 Microbiology Institute, Catholic University of the Sacred Heart (UCSC), Largo Francesco Vito 1, 00168 Rome, Italy; 4 Department of Physics, University Sapienza, Piazzale Aldo Moro 5, 00185 Rome, Italy; 5 Department of Physical and Chemical Sciences, University of L’Aquila, Via Vetoio 10, I-67010 L’Aquila, Italy

## Abstract

A correction to this article has been published and is linked from the HTML version of this paper. The error has not been fixed in the paper.

*Scientific Reports* **6**:12; doi:10.1038/s41598-016-0010-7; Article published online 05 December 2016

This Article contains a typographical error in the Methods section, under the subheading “Growing cell simulation model”, where:

“When the *i*-th cell has doubled its length it splits into two equal cells *i*_1_ and *i*_2_ of length ${\ell }_{0}$, located at ${{\bf{r}}}_{{i}_{1},{i}_{2}}={{\bf{r}}}_{i}\pm {\hat{{\bf{e}}}}_{i}{\ell }_{0}/4$ and with orientation ${{\bf{e}}}_{{i}_{1},{i}_{2}}={\ell }_{0}{\mathscr{R}}[{\hat{{\bf{e}}}}_{i}]$”.

should read:

“When the *i*-th cell has doubled its length it splits into two equal cells *i*_1_ and *i*_2_ of length ${\ell }_{0}$, located at ${{\bf{r}}}_{{i}_{1},{i}_{2}}={{\bf{r}}}_{i}\pm {\hat{{\bf{e}}}}_{i}{\ell }_{0}/2$ and with orientation ${{\bf{e}}}_{{i}_{1},{i}_{2}}={\ell }_{0}{\mathscr{R}}[{\hat{{\bf{e}}}}_{i}]$”.

