# Supplementary material for: Biomimetic antimicrobial cloak by graphene-oxide agar hydrogel
Source: Sci Rep. 2016 Dec 5;6:12. doi: 10.1038/s41598-016-0010-7 (PMC5431354; doi:10.1038/s41598-016-0010-7)
Supplement: Supplementary file 1 — Supplementary Information [file 41598_2016_10_MOESM1_ESM.doc]

# Biomimetic antimicrobial cloak by graphene-oxide agar hydrogel

**Authors**

**Massimiliano Papi^1,3*^, Valentina Palmieri^1,3*^, Francesca Bugli^2^, Marco De Spirito^1^, Maurizio Sanguinetti^2^**

**Carlotta Ciancico^3,4^, Maria Chiara Braidotti^3,5^, Silvia Gentilini^3,4^, Luca Angelani^3,4^, Claudio Conti^3,4^**

**Affiliations:**

1. Physics Institute, Catholic University of the Sacred Heart (UCSC), Largo Francesco Vito 1, 00168 Rome (IT).
2. Microbiology Institute, Catholic University of the Sacred Heart (UCSC), Largo Francesco Vito 1, 00168 Rome (IT).
3. Institute for Complex Systems, National Research Council (ISC-CNR), Via dei Taurini 19, 00185 Rome (IT).
4. Department of Physics, University Sapienza, Piazzale Aldo Moro 5, 00185 Rome (IT).
5. Department of Physical and Chemical Sciences, University of L'Aquila, Via Vetoio 10, I-67010 L’Aquila (IT).

***These authors equally contributed equally to this work**

# Supplementary Information

# Details of exposing nanosheet in GO


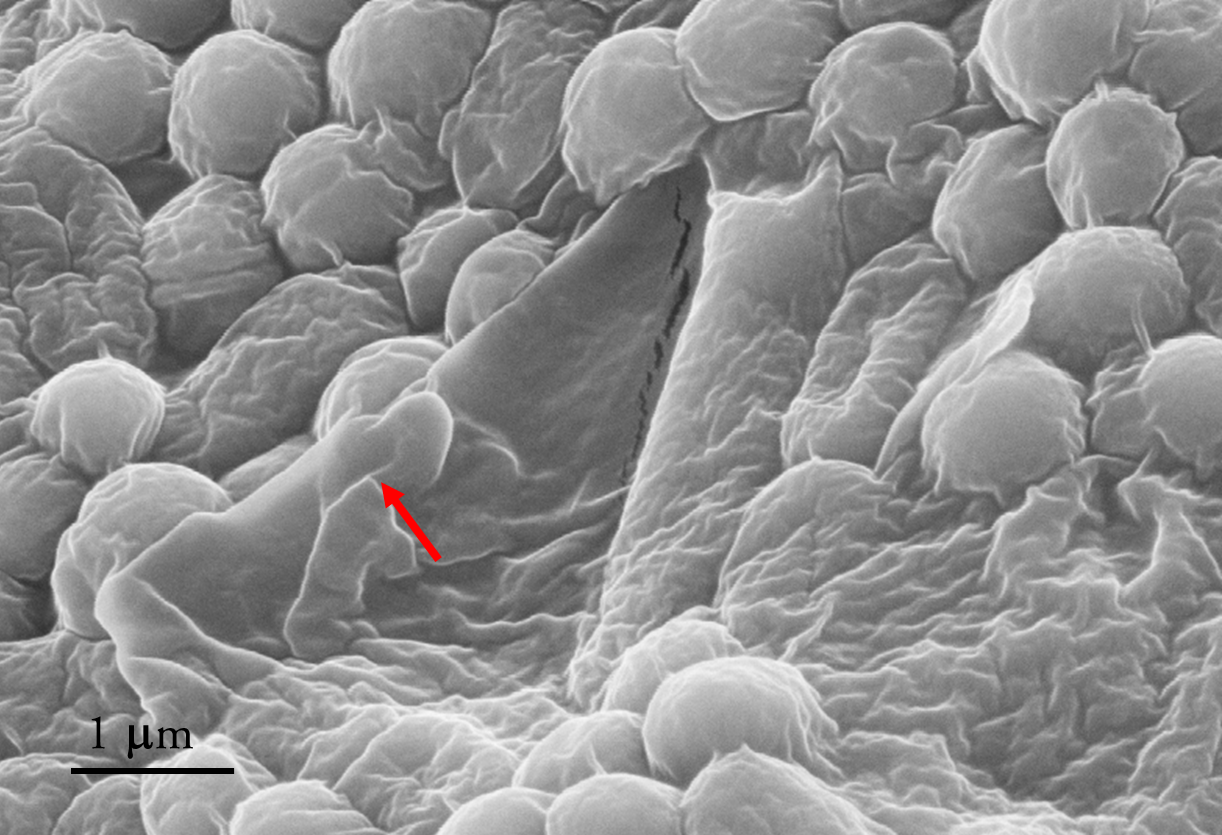


**Fig. S1** GO sheets emerging from the surface. S. aureus debris after membrane cutting are highlighted with a red arrow.

## Hydrogel microporosity


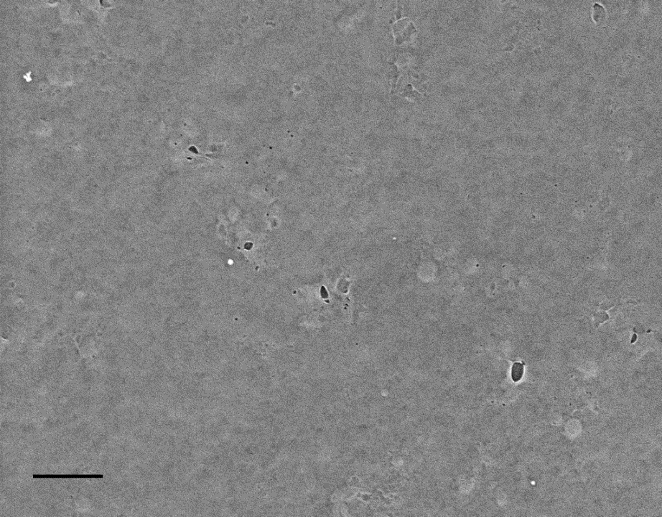

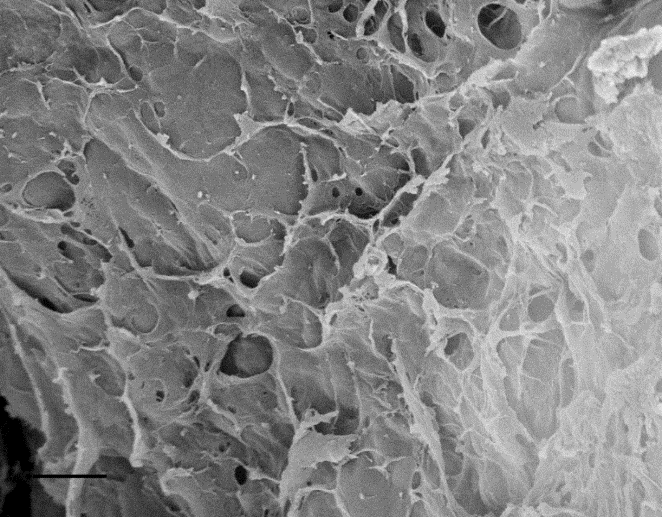


**1** μ**m**

**1** μ**m**

**A**

**B**

**Fig. S2** High resolution details of the smooth unpatterned GO surface (A) and of the GO patterned surface (B).
